# Advances in Lithium–Oxygen Batteries Based on Lithium Hydroxide Formation and Decomposition

**DOI:** 10.3389/fchem.2022.923936

**Published:** 2022-07-01

**Authors:** Xiahui Zhang, Panpan Dong, Min-Kyu Song

**Affiliations:** School of Mechanical and Materials Engineering, Washington State University, Pullman, WA, United States

**Keywords:** lithium–oxygen batteries, LiOH chemistry, reversibility, high energy batteries, reaction mechanisms, redox mediator, lithium metal anode

## Abstract

The rechargeable lithium-oxygen (Li–O_2_) batteries have been considered one of the promising energy storage systems owing to their high theoretical energy density. As an alternative to Li−O_2_ batteries based on lithium peroxide (Li_2_O_2_) cathode, cycling Li−O_2_ batteries *via* the formation and decomposition of lithium hydroxide (LiOH) has demonstrated great potential for the development of practical Li−O_2_ batteries. However, the reversibility of LiOH-based cathode chemistry remains unclear at the fundamental level. Here, we review the recent advances made in Li−O_2_ batteries based on LiOH formation and decomposition, focusing on the reaction mechanisms occurring at the cathode, as well as the stability of Li anode and cathode binder. We also provide our perspectives on future research directions for high-performance, reversible Li−O_2_ batteries.

## Introduction

**FIGURE 1:**
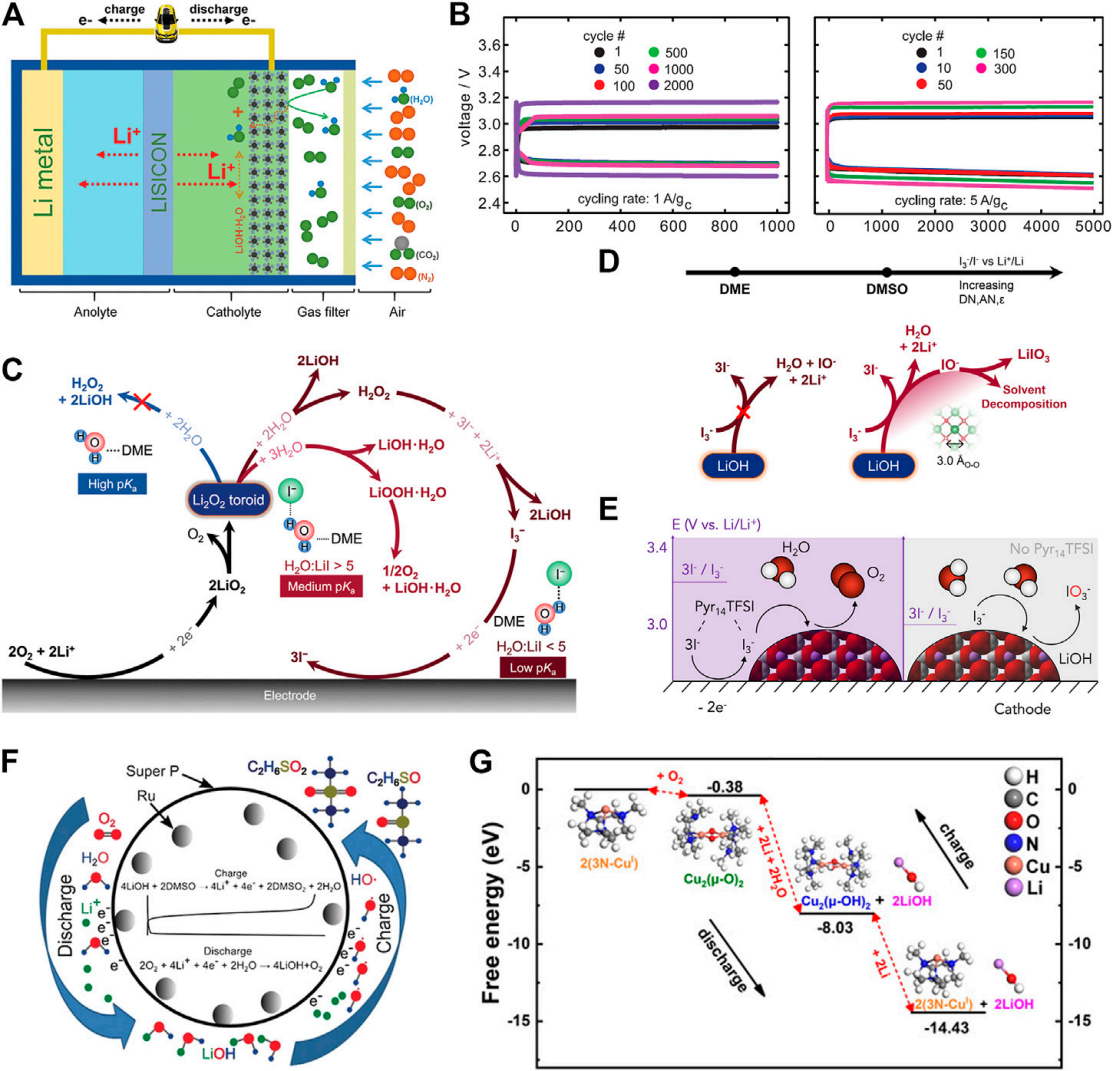
Representative Li−O_2_ batteries based on LiOH formation and decomposition. **(A)** Schematic illustration of Li−air batteries based on LiOH chemistry (Wu et al., 2016). **(B)** Cycling performance of Li−air batteries based on LiOH chemistry (Liu et al., 2015). **(C)** Reaction pathways of the formation of LiOH in LiI-containing electrolytes (Tulodziecki et al., 2017). **(D)** Solvent-dependent oxidation power of $I_{3}^{-}/I^{-}$ redox couple in DME or DMSO toward the oxidation of LiOH (Leverick et al., 2019). **(E)** Reversible oxidation of LiOH by $I_{3}^{-}/I^{-}$ redox couple in Pyr_14_TFSI/TEGDME electrolyte (Temprano et al., 2020). **(F)** Irreversible oxidation of LiOH by Ru catalyst (Liu et al., 2017). **(G)** Energy diagram of reversible oxidation of LiOH by 3N-Cu^Ⅰ^ complex by DFT calculations (Wang et al., 2020).

Lithium–oxygen (Li−O_2_) batteries, also known as Li–air batteries, use lithium metal as an anode and Earth-abundant O_2_ as a cathode-active material (Figure 1A) (Aurbach et al., 2016). In general, Li−O_2_ batteries can be divided into two categories, depending on the type of electrolytes that separate cathode from anode, (ⅰ) aqueous and (ⅱ) non-aqueous Li−O_2_ batteries. Owing to the high reactivity of water toward Li metal anode, most studies focus on non-aqueous Li−O_2_ batteries. Non-aqueous Li−O_2_ batteries were first demonstrated by Abraham and Jiang (1996) using a polymer-based electrolyte. Since then, the research and development of Li−O_2_ batteries have blossomed (Aurbach et al., 2016; Chang et al., 2017; Lim et al., 2017; Song et al., 2017). Three types of battery chemistries have been reported, including 1e^−^ lithium superoxide (LiO_2_) (Lu et al., 2016), 2e^−^ lithium peroxide (Li_2_O_2_) (McCloskey et al., 2013), and 4e^−^ lithium oxide (Li_2_O) and lithium hydroxide (LiOH) (Liu et al., 2015; Lei et al., 2022). The corresponding reactions are summarized below.

$$O_{2}+Li^{+}+e^{-}\to \mathrm{Li}O_{2},E^{0}=3.0V\left(\mathrm{vs}.Li^{+}/\mathrm{Li}\right),$$ (1)

$$O_{2}+2Li^{+}+2e^{-}\to Li_{2}O_{2},E^{0}=2.96V\left(\mathrm{vs}.Li^{+}/\mathrm{Li}\right),$$ (2)

$$O_{2}+4Li^{+}+4e^{-}\to 2Li_{2}O,E^{0}=2.91V(\mathrm{vs}.Li^{+}/\mathrm{Li}),$$ (3)

$$O_{2}+2H_{2}O+4Li^{+}+4e^{-}\to 4\mathrm{LiOH},E^{0}=3.32V\left(\mathrm{vs}.Li^{+}/\mathrm{Li}\right).$$ (4)

Li_2_O_2_ is the most reported discharge product in non-aqueous Li−O_2_ batteries because, under ambient conditions, LiO_2_ is thermodynamically unstable against disproportionation (Zhang et al., 2017), and Li_2_O is thermodynamically unfavorable owing to a lower electrode potential of *E* ^0^(O_2_/Li_2_O) = 2.91 V. It is also noted that Li_2_O can be thermodynamically more favorable over Li_2_O_2_ as discharge product in a molten-salt electrolyte at >150°C (Xia et al., 2018), which is beyond the scope of this study. When compared with Li_2_O_2_ chemistry, Li−O_2_ batteries based on LiOH chemistry can be operated in humid environments and demonstrate better resistance toward CO_2_ (Lei et al., 2022), which might enable the operation of Li−O_2_ batteries in the air. Liu et al. (2015) reported high-performance Li−O_2_ batteries based on LiOH formation and decomposition *via* iodide catalysis (Figure 1B). Since then, many research efforts have been devoted to LiOH chemistry (Table 1), which significantly advances the understanding of this chemistry.

**TABLE 1:** Summary of representative LiOH chemistry in Li−O_2_ batteries.

Cathode^a^	Electrolyte^b^	Water source^c^	Catalyst	Discharge products	O_2_ evolution	Reference
GDL	1 M LiI in G4	Electrolyte degradation	I^−^	LiOH	-	Kwak et al. (2015)
rGO^d^	0.05 M LiI + 0.25 M LiTFSI in DME	-	I^−^	LiOH	Yes	Liu et al. (2015)
SP	0.2 M LiI + 0.05 M LiTFSI in DME	500 ppm H_2_O in DME	I^−^	Li_2_O_2_ + LiOH	Yes^e^	Burke et al. (2016)
Carbon felt	0.05 M LiI +0.5 M LiTFSI in DME	9.1% H_2_O	I^−^	LiHO_2_·H_2_O + LiOH·H_2_O	Yes^f^	Zhu et al. (2017)
KB	1 M LiI in DME	Electrolyte degradation	I^−^	LiOH	No	Qiao et al. (2017)
VC	0.1 M LiI + 0.5 M LiTFSI in G2 or DMSO	-	-	Pre-loaded LiOH	No	Leverick et al. (2019)
Ensaco-P150	0.05 M LiI + 0.7 M LiTFSI + 0.9 M Pyr_14_TFSI in G4	5000 ppm H_2_O in G4	I^−^	LiOH	Yes	Temprano et al. (2020)
Carbon paper	0.5NaTFSI + 1 M LiTFSI in G4	Electrolyte degradation	Na^+^	LiOH	Yes	Bi et al. (2020)
KB	0.05 M 3N-Cu^Ⅰ^ + 1 M LiTFSI in G4	2000 ppm H_2_O in DME	3N-Cu^Ⅰ^	LiOH	Yes	Wang et al. (2020)
Ru/MnO_2_/SP	0.5 M LiClO_4_ in DMSO	120 ppm H_2_O in DMSO	γ-MnO_2_	Li_2_O_2_ + LiOH	Yes	Peng et al. (2015)
Ru/MnO_2_/SP	0.5 M LiTFSI in [pmim][TFSI]	RH 51% in O_2_	γ-MnO_2_	LiOH	-	Wu et al. (2016)
Ru/CNT	1.5 M LiNO_3_ in DMSO	62 ppm H_2_O in DMSO	Ru	Li_2_O_2_ + LiOH	Yes	Sun et al. (2016)
Ru/SP	1 M LiTFSI in DMSO	4000 ppm H_2_O in DMSO	Ru	LiOH	No	Liu et al. (2017)
MnCo-MOF-74/KB	1 M LiTFSI in G4	-	Mn-MOF-74	LiOH	-	Kim et al. (2018)
Mn-MOF-74@CNT/KB	1 M LiTFSI in G4	200 ppm H_2_O in O_2_	Mn-MOF-74	LiOH	Yes	Zhang et al. (2019)
AG^[d]^	1 M LiTFSI in G4	H_2_O in AG	-	LiOH	Yes	Mu et al. (2019)
Co_3_O_4_	0.25 M LiTFSI in G2	5% H_2_O in G2	Co_3_O_4_	LiOH	Yes	Lu et al. (2020)
Ru/SP	1 M LiTFSI in DMSO	5% H_2_O in DMSO	Ru	LiOH	No	Lei et al. (2022)
Ru/SP	1 M LiTFSI in DMSO	5% H_2_O in DMSO	Ru	LiOH	No	Tang et al. (2022)
Ag/δ-MnO_2_	1 M LiTFSI in G4	H_2_O in Ag/δ-MnO_2_	δ-MnO_2_	LiOH	-	Dai et al. (2022)

a GDL, gas diffusion layer; rGO, reduced graphene oxide; SP, super-P; KB, ketjenblack; VC, Vulcan carbon; Ensaco-P150, a mesoporous carbon black; AG, activated graphene; CNT, carbon nanotube.

b TFSI, bis(trifluoromethylsulfonyl)imide; DME (G1), 1,2-dimethoxyethane; DEGDME (G2), diethylene glycol dimethyl ether; TEGDME (G4), tetraethylene glycol dimethyl ether; DMSO, dimethyl sulfoxide; Pyr_14_TFSI, 1-butyl-1-methylpyrrolidinium bis(trifluoromethylsulfonyl)imide; [pmim][TFSI], 1-methyl-3-propylimidazolium bis(trifluoromethylsulfonyl)imide; RH, relative humidity.

c The water source claimed by the authors of that reference at that time.

d We note that the preparation of rGO and AG uses KMnO_4_, which may result in Mn impurities (Wang et al., 2019) in rGO and AG for the catalytic formation of LiOH.

e O_2_ evolution observed from the decomposition of Li_2_O_2_ rather than LiOH.

f O_2_ evolution observed from the decomposition of LiOH by I_2_ in DME/H_2_O (10/1, v/v).

Here, we present an overview of Li−O_2_ batteries based on the formation and decomposition of LiOH. This review begins with the fundamentals of the formation of LiOH, followed by the decomposition of LiOH. Furthermore, we discuss the stability of Li metal anode and cathode binder in brief. Finally, we provide the outlook on how to achieve reversible LiOH chemistry for advanced Li−O_2_ batteries.

## Fundamentals of Lithium Hydroxide Formation at the Cathode

### Reported Mechanisms on Discharge

The formation of LiOH in Li−O_2_ batteries is based on 4e^−^ O_2_ reduction, which renders this process complicated. The first step involves O_2_ reduction to form a superoxide intermediate, that is, LiO_2_ or O_2_^−^ (Eq. 5). In the following steps, several reaction intermediates and pathways have been reported in the literature, as summarized below.

$$O_{2}+Li^{+}+e^{-}\to \mathrm{Li}O_{2}.$$ (5)

1) Direct hydrolysis of LiO_2_ (Wang et al., 2017; Bi et al., 2020).

$$4\mathrm{Li}O_{2}+2H_{2}O\to 4\mathrm{LiOH}+3O_{2}.$$ (6)

2) Hydrolysis of LiO_2_ to form LiHO_2_ intermediate, followed by disproportionation of LiHO_2_ (Qiao et al., 2017).

$$2\mathrm{Li}O_{2}+H_{2}O\to \mathrm{LiOH}+\mathrm{LiH}O_{2}+O_{2},$$ (7)

$$2\mathrm{LiH}O_{2}\to 2\mathrm{LiOH}+O_{2}.$$ (8)

3) Disproportionation of LiO_2_ to form Li_2_O_2_ intermediate, followed by hydrolysis of Li_2_O_2_ (Peng et al., 2015; Tulodziecki et al., 2017).

$$2\mathrm{Li}O_{2}\to Li_{2}O_{2}+O_{2},$$ (9)

$$Li_{2}O_{2}+2H_{2}O\to \mathrm{2LiOH}+H_{2}O_{2},$$ (10)

$$2H_{2}O_{2}\to 2H_{2}O+O_{2}.$$ (11)

The total reaction for each of the abovementioned three reaction pathways is the 4e^−^ O_2_ reduction (Eq. 4), leading to LiOH as a discharge product. The reaction pathways depend on several factors, such as electrolytes, water content, and catalysts. The source of water and the role of catalysts are discussed in the following subsections. Representative LiOH systems are summarized in Table 1.

### Source of Proton: Wet Electrolyte, Humid O_2_, or Water-Trapped Electrode

The formation of LiOH involves the hydrolysis of reaction intermediates, which requires the addition of water in Li−O_2_ cells. Water has been commonly introduced into either electrolyte (wet electrolyte) or O_2_ gas (humid O_2_). In early studies, however, the formation of LiOH was observed even in nominal dry electrolyte and dry O_2_ (Liu et al., 2015). This observation raises the question of where the proton comes from. In early studies, Kwak et al. (2015) and Qiao et al. (2017) reported that the source of the proton is the H-abstraction of electrolyte solvent, leading to electrolyte degradation and water formation. Later, Liu et al. (2019) demonstrated that the source of the proton is the water impurity in the electrolyte using isotope-labeled solid-state nuclear magnetic resonance (NMR) and Raman spectroscopy. We note that water impurity can be either from lithium salt or solvent, and thus the measurement of water content in electrolytes is highly recommended. Moreover, theoretical calculations also reveal that water is a much more favorable proton source than the electrolyte solvent (DME) (Torres and Balbuena, 2018). In addition to the water impurity in the electrolyte, water can be trapped in the cathode. Mu et al. (2019) reported that an activated graphene electrode can adsorb enough water (4.56%) for the formation of LiOH. A similar observation was also reported in the Ag/δ-MnO_2_ electrode (Dai et al., 2022).

We note that water might also come from the moisture in leaking cells, which is a common issue that causes the discrepancy in discharge capacity and morphology of discharge products in reported Li−O_2_ batteries. In leaking-free dry cells, film-like discharge products should be observed in low-donor-number and low-acceptor-number electrolytes (such as DME) (Aetukuri et al., 2015), which is a simple method to check if any leakage exists.

### Role of Catalyst: Promotion of the Cleavage of O−O Bond

Unlike Li_2_O_2_, the formation of LiOH involves the cleavage of strong O−O bond (Eqs 8 and 11), which requires an effective catalyst to lower activation energy. Two types of catalysts have been widely studied, including soluble catalyst in the electrolyte and solid catalyst on the cathode. Representative catalysts are summarized in Table 1.

Soluble catalyst is also known as a redox mediator in Li−O_2_ batteries, and LiI is one of the most promising catalysts. Kwak et al. (2015) reported that LiI can facilitate the formation of LiOH *via* a proposed mechanism of iodide catalysis (Eqs 12 and 13).

$$HO_{2}^{-}+I^{-}\to OH^{-}+IO^{-},$$ (12)

$$HO_{2}^{-}+IO^{-}\to OH^{-}+O_{2}+I^{-}.$$ (13)

Qiao et al. (2017) successfully detected the reaction intermediates of $HO_{2}^{-}$ and IO^−^ by Raman and UV-Vis spectroscopy, respectively, which confirms the abovestated mechanism of iodide catalysis for the formation of LiOH. Moreover, Qiao et al*.* observed that the catalytic activity of iodide decreases with the increase of water content in the electrolyte, leading to the formation of a Li_2_O_2_/LiOH mixture. This decrease in catalytic activity was ascribed to the high concentration of OH^−^ ions in water-rich electrolyte that pushes the equilibrium of Eqs 12 and 13 to the left, that is, sluggish iodide catalysis. Later, Tulodziecki et al. (2017) demonstrated that iodide catalysis involves $I^{-}/I_{3}^{-}$ couple *via* Eqs 14 and 15 (Figure 1C) using Raman and UV-Vis spectroscopy rather than I^−^/IO^−^ couple.

$$H_{2}O_{2}+3I^{-}\to I_{3}^{-}+2OH^{-},$$ (14)

$$I_{3}^{-}+2e^{-}\to 3I^{-}.$$ (15)

Moreover, Tulodziecki et al*.* further demonstrated that low p*K*_a_ of water in electrolyte facilitates Eq. 10 and, thus, is essential to the formation of LiOH. In LiI-containing electrolytes, high water content leads to high p*K*a of water (lower acidity) and thus sluggish hydrolysis of Li_2_O_2_ to H_2_O_2_ (Eq. 10). Overall, this lower acidity of water leads to apparent sluggish iodide catalysis and, thus, the formation of a Li_2_O_2_/LiOH mixture. Later, Liu et al. (2019) proposed the third explanation for sluggish iodide catalysis. In water-rich electrolytes (>5% H_2_O), I^−^ ions are embedded in I^−^(H_2_O)_*n*_ ion clusters, as revealed by MD simulations, which makes it difficult to be accessible for iodide catalysis, leading to sluggish iodide catalysis and, thus, the formation of Li_2_O_2_/LiOH mixture. In addition to Eq. 15, Liu et al. observed an alternative path for regeneration of I^−^ from $I_{3}^{-}$ in water-rich electrolytes *via* chemical reaction (Eq. 16).

$$I_{3}^{-}+H_{2}O_{2}+2OH^{-}\to 3I^{-}+2H_{2}O+O_{2}.$$ (16)

Wang et al. (2020) reported a bio-inspired 3N-Cu^Ⅰ^ enzyme as a soluble catalyst for LiOH chemistry, which is effective for the cleavage of O−O bond (Figure 1G). The reaction intermediate is detected as Cu_2_(μ-O)_2_ complex by UV-Vis spectroscopy.

In addition to the abovementioned soluble catalysts, solid catalysts on the cathode have been demonstrated to be effective for the formation of LiOH. Peng et al. (2015) reported Ru@SP/MnO_2_ catalyst for LiOH chemistry, where MnO_2_ is effective for catalytic decomposition of H_2_O_2_ (Eq. 11). Since then, a few other cathode catalysts have been reported, such as Mn-MOF-74, Co-MOF, and Co_3_O_4_ (Kim et al., 2018; Yuan et al., 2019; Zhang et al., 2019; Lu et al., 2020). In our previous study, the authors reported that the Mn-MOF-74 catalyst was still active even if it was coated on a separator that was electrically isolated from the cathode (Zhang et al., 2019), demonstrating that the formation of LiOH can proceed *via* chemical hydrolysis of reaction intermediates.

## Fundamentals of Lithium Hydroxide Decomposition at the Cathode

### Reported Mechanisms on Charge

The decomposition of LiOH on charge is much more complex but less explored than the formation of LiOH. This process varies with catalyst and electrolyte, and it usually involves irreversible parasitic reactions. Some reported mechanisms are summarized below.

1) O_2_ evolution with a solid catalyst, for example, Ru and Co_3_O_4_ (Peng et al., 2015; Lu et al., 2020).

$$4\mathrm{LiOH}\to 2H_{2}O+O_{2}+4Li^{+}+4e^{-}.$$ (17)

2) Irreversible formation of hydroxyl radical (OH·) that degrades electrolyte with a solid catalyst (Liu et al., 2017; Tang et al., 2022).

$$\mathrm{LiOH}\to \mathrm{OH}\cdot +Li^{+}+e^{-},$$ (18)

$$2\mathrm{OH}\cdot +\mathrm{DMSO}\to \mathrm{DMS}O_{2}+H_{2}O.$$ (19)

3) O_2_ evolution with redox mediator, for example, LiI (Liu et al., 2015; Temprano et al., 2020).

$$3I^{-}\to I_{3}^{-}+2e^{-},$$ (20)

$$4\mathrm{LiOH}+2I_{3}^{-}\to 2H_{2}O+O_{2}+4Li^{+}+6I^{-}.$$ (21)

4) Irreversible formation of IO_3_^−^ after Eq. 20 in LiI-containing electrolyte (Leverick et al., 2019).

$$6\mathrm{LiOH}+3I_{3}^{-}\to 3H_{2}O+6Li^{+}+IO_{3}^{-}+8I^{-}.$$ (22)

Given the complexity of LiOH chemistry, systematic studies are needed to confirm its reversibility, which is discussed in the following subsections.

### Reversibility and Parasitic Reactions

The reversibility of LiOH chemistry is defined as how much O_2_ evolves back to air during charge, which is different from “rechargeability” that typically features by the removal of discharge product. We emphasize that the removal of LiOH after charge does not guarantee good reversibility because it might be due to parasitic reactions (Eqs 19 and 22).

In early studies, Meini et al. (2013) demonstrated that pre-loaded LiOH cannot be decomposed to O_2_ with or without Pt catalyst in diglyme electrolyte by differential electrochemical mass spectroscopy (DEMS). Moreover, theoretical calculations suggest that oxidation of LiOH needs high overpotential and is controlled by the extraction of Li^+^ to form an O-rich surface at 4.6–5.0 V (vs. Li^+^/Li) (Ling et al., 2014). Later, Peng et al. (2015) reported that LiOH can be decomposed to O_2_ by Ru catalyst in DMSO electrolyte *via* Eq. 17, as revealed by gas chromatography (GC). However, Liu et al. (2017) demonstrated that oxidation of LiOH in DMSO electrolyte evolves little O_2_ using DEMS and operando cell pressure measurements, revealing the irreversibility of LiOH chemistry. This irreversibility was ascribed to the formation of hydroxyl radical that attacks the DMSO electrolyte *via* Eqs 18 and 19 (Figure 1F). Tang et al. (2022) further detected hydroxyl radical using a radical trap by *in situ* UV-Vis spectroscopy. Yang et al. (2016) also revealed the irreversibility of pre-loaded Li^18^OH in DMSO electrolytes by isotope-labeled DEMS. We note that pre-loaded LiOH typically shows a high charge potential at ∼5 V (vs. Li^+^/Li), at which electrolyte might undergo severe electromechanical oxidation.

In addition to solid catalysts, soluble catalysts have also been reported to facilitate the reversible oxidation of LiOH. In early studies, Liu et al. (2015) reported that LiOH can be decomposed to O_2_ by iodide catalysis at ∼3.0 V (vs. Li^+^/Li) in DME-based electrolytes *via* Eqs 20 and 21, as revealed by mass spectrometry. However, Viswanathan et al. (2016) argued that the oxidation of LiOH to O_2_ (*E* ^0^ = 3.34 V vs. Li^+^/Li) by $I_{3}^{-}/I^{-}$ redox couple at ∼3.0 V (vs. Li^+^/Li) is thermodynamically unfavorable. Alternatively, Burke *et al.* (2016) demonstrated that LiOH might be oxidized to LiIO_3_ by $I_{2}/I_{3}^{-}$ redox couple at ∼3.5 V (vs. Li^+^/Li) *via* Eqs 23 and 24, leading to a lack of O_2_ evolution as revealed by DEMS.

$$2I_{3}^{-}\to 3I_{2}+2e^{-},$$ (23)

$$6\mathrm{LiOH}+3I_{2}\to 3H_{2}O+6Li^{+}+IO_{3}^{-}+5I^{-}.$$ (24)

Shen et al. (2016) and Qiao et al. (2017) demonstrated that LiOH cannot be oxidized by $I_{3}^{-}/I^{-}$ redox couple in DME electrolyte, as revealed by XRD and Raman spectroscopy. In response, Liu et al. (2016) confirmed the formation of IO_3_^−^ by UV-Vis spectroscopy. Nonetheless, they still argued that the removal of LiOH after the charge was indeed observed by SEM and XRD, and the oxidation of LiOH to O_2_ by $I_{3}^{-}/I^{-}$ redox couple might be thermodynamically favorable if considering the solvation effect and low activity of water. Interestingly, Leverick et al. (2019) demonstrated that the oxidation power of $I_{3}^{-}/I^{-}$ redox couple is indeed affected by the solvation effect (Figure 1D). They observed that an electrolyte solvent having higher solvation power (DMSO) leads to the higher oxidation power of $I_{3}^{-}/I^{-}$ redox couple when compared with that in a solvent with low solvation power (DME). This observation explains that LiOH can be oxidized by $I_{3}^{-}/I^{-}$ redox couple in DMSO *via* Eq. 22, whereas it cannot be oxidized in DME (Figure 1D). We note that water has high solvation power owing to its high AN of 54.8, Aetukuri et al. (2015) and, thus, $I_{3}^{-}/I^{-}$ redox couple might have enough oxidation power toward oxidation of LiOH *via* Eq. 22 in water-containing DME electrolyte.

### Routes Toward Reversible Lithium Hydroxide Chemistry

The reversible 4e^−^ oxidation of LiOH to O_2_ involves the reformation of O−O bond, which generates many reactive intermediates (reactive oxygen species). This process is feasible in an aqueous solution but is very challenging in organic electrolytes, owing to the instability of organic electrolytes toward these reactive intermediates. Therefore, less-reactive intermediates or more stable electrolytes are required to achieve reversible LiOH chemistry.

Given that amorphous Li_2_O_2_ may be formed simultaneously with LiOH on discharge, regular DEMS cannot differentiate from which O_2_ is evolved: Li_2_O_2_ or LiOH. As such, the isotope-labeled DEMS is recommended to exclusively determine whether LiOH is reversibly oxidized to O_2_ on charge. For experimental designs, isotope-labeled DEMS can be achieved by ether labelling O_2_ (^18^O_2_ or ^17^O_2_) or labelling H_2_O (H_2_^17^O or H_2_^18^O). An example of labelling H_2_O by H_2_^17^O is shown in Eqs 25 and 26. If LiOH can be reversibly oxidized to O_2_, a mixture of ^16^O_2_, ^16^O^17^O, and ^17^O_2_ should be observed using DEMS.

$${}^{16}O_{2}+2H_{2}^{17}O+4Li^{+}+4e^{-}\to 2Li^{16}OH+2Li^{17}OH,$$ (25)

$$2Li^{16}OH+2Li^{17}OH\to 0.25^{16}O_{2}+0.5^{16}O^{17}O+0.25^{17}O_{2}+H_{2}^{16}O+H_{2}^{17}O+4Li^{+}+4e^{-},$$ (26)

Recently, Temprano et al. (2020) reported that truly reversible oxidation of LiOH was achieved by $I_{3}^{-}/I^{-}$ redox couple in Pyr_14_TFSI/TEGDME electrolyte, as revealed by isotope-labeled DEMS (H_2_^17^O) and operando cell pressure measurements. They claimed that Pyr_14_TFSI, one kind of ionic liquid, is essential for achieving reversible O_2_ evolution by tuning the oxidation powder of $I_{3}^{-}/I^{-}$ redox couple (Figure 1E). This finding suggests that the redox mediator might facilitate reversible oxidation of LiOH by tuning the solvation structure of active species and, thus, oxidation power of the redox mediator. However, Kim et al. (2020) reported that TEMPO with a high redox potential of 3.74 V (vs. Li^+^/Li) could not decompose LiOH, revealing that the oxidation power of a redox mediator is not the only limiting factor for achieving the reversible oxidation of LiOH. Therefore, we suggest that more systematic studies are still needed to understand how the 4e^−^ O_2_ evolution reaction by $I_{3}^{-}/I^{-}$ redox couple proceeds *via* Eq. 21.

Recently, Lu et al. (2020) reported that the Co_3_O_4_ catalyst can facilitate the reversible oxidation of LiOH to evolve O_2,_ as revealed by isotope-labeled DEMS (H_2_^17^O). This new finding suggests that the catalyst governs the reactivity of intermediates during the oxidation of LiOH, although more systematic studies are needed to understand why reactive hydroxyl species were not formed in this system. Wang et al. (2020) reported that reversible oxidation of LiOH was achieved by bio-inspired 3N-Cu^Ⅰ^ complex (Figure 1G), as revealed by DEMS and DFT calculations. The reversible LiOH chemistry was attributed to the effective cleavage and reformation of O−O bond by 3N-Cu^Ⅰ^ complex. However, we note that reactive oxygen species is embedded in the 3N-Cu^Ⅰ^ complex (Figure 1G), which might make it difficult to attack organic electrolyte, leading to reversible oxidation of LiOH to O_2_. In addition, we note that the 3N-Cu^Ⅰ^ complex was prepared by CuI, and thus the role of I^−^ anions should be carefully studied in the future. Nonetheless, this new finding demonstrates the great potential of using bio-inspired complex to deactivate reactive oxygen species, which deserves more effort to explore.

## Li Metal Anode and Cathode Binder

### Stability of Li Anode

Lithium metal anodes have been studied intensely since the 1970s, owing to their ultralow reduction potential of −3.04 V (vs. SHE) and ultrahigh specific capacity of 3,860 mAh g^−1^ (Cheng et al., 2017). However, their practical application has been hindered owing to (ⅰ) safety concerns caused by dendritic lithium growth and (ⅱ) poor Coulombic efficiency caused by dead lithium and continuous solid electrolyte interphase (SEI) formation (Lu et al., 2011; Qian et al., 2015). During repeated Li plating/stripping processes, the huge mechanical stress generated from the infinite volume change of lithium results in the cracking of the SEI layer, leading to continuous SEI formation and dendritic lithium growth. Moreover, the large concentration gradient of Li^+^ ions at high current density can significantly accelerate dendritic lithium growth, leading to safety hazards. Substantial research effort has been devoted to stabilizing the lithium metal surface using electrolyte additives and/or an artificial SEI layer. In addition, solid-state electrolytes have emerged to address safety concerns by eliminating flammable organic liquid electrolytes (Manthiram et al., 2017). Recently, it has been reported that cycling Li metal in O_2_ is much more stable when compared with that in argon, suggesting that O_2_ is good for robust SEI formation (Qiu et al., 2018). Furthermore, cycling Li metal in CO_2_ could generate a robust Li_2_CO_3_-rich SEI layer, leading to a dendrite-free and moisture-tolerant Li metal anode (Asadi et al., 2018; Chen et al., 2020; Qiu et al., 2020).

Owing to its high reactivity, Li metal anode can severely react with water or some redox mediators in Li−O_2_ batteries (Guo et al., 2014), leading to parasitic reactions. Therefore, protection of Li metal anode is needed for LiOH chemistry. Recently, water-proof Li metal anodes have been developed, such as wax-infiltrated solid-state electrolyte layer (Wu et al., 2017; Lei et al., 2022). Besides the protection of Li anode, alternative reference electrodes have been developed for studying the effect of water or redox mediators on battery chemistry and performance. The lithium iron phosphate (LiFePO_4_) electrode has a redox potential of 3.45 V (vs. Li^+^/Li) with fast reaction kinetics, and thus has been widely used as alternative reference anode when Li metal anode is unstable (Li et al., 2015; Peng et al., 2015; Mahne et al., 2017; Ma et al., 2018). However, it has been reported that LiFePO_4_ electrode still could electrochemically react with some redox mediators such as tetrathiafulvalene (TTF) (Yang et al., 2016). Therefore, the reactivity of reference electrode with cell components should be carefully examined when selecting a reference electrode.

### Stability of Cathode Binders: Polyvinylidene Fluoride and Polytetrafluoroethylene

A binder is essential for holding active materials on current collect when fabricating electrodes. Two binders are commonly used in Li−O_2_ batteries, polyvinylidene fluoride (PVdF) and polytetrafluoroethylene (PTFE). It has been reported that PVdF is unstable toward LiO_2_ and LiOH, leading to defluorination (Black et al., 2012; Papp et al., 2017). Therefore, the PTFE binder is encouraged in studies for LiOH chemistry.

## Summary and Outlook

LiOH chemistry is promising for advanced Li−O_2_ batteries that can be operated in humid environments, considering that the requirements for removing moisture from the air down to a few ppm levels in conventional Li−O_2_ batteries (based on Li_2_O_2_) not only increase the cost but also reduce energy density (Gallagher et al., 2014). Moreover, cycling Li−O_2_ batteries based on LiOH chemistry has demonstrated great energy efficiency and rate capability (Figure 1B) because of soluble intermediates in wet electrolytes and mitigated passivation of the cathode. Since 2015, many research efforts have been devoted to LiOH chemistry. During discharge, the formation of LiOH involves the cleavage of O−O bond by the catalyst and the hydrolysis of reaction intermediates. During charge, the decomposition of LiOH is very complex because of parasitic reactions. The reversible oxidation of LiOH involves the reformation of O−O bond, which may generate many reactive oxygen species that attack organic electrolytes, including redox mediators. In early studies, many reports demonstrated that LiOH chemistry is irreversible in organic electrolytes and O_2_ evolution is lacking during charge. Markedly, a few recent studies have demonstrated that reversible LiOH chemistry can be achieved by tuning the oxidation power of the redox mediator or deactivating the reactive intermediates. Nonetheless, LiOH chemistry is still at an infant stage. There is still a lack of fundamental understanding of reaction mechanisms, which is essential for achieving truly reversible LiOH chemistry. In addition, most studies by far were performed in ultrapure O_2_, which is not practical for commercial applications of Li−O_2_ batteries. Moreover, the use of Li metal anode in Li−O_2_ batteries may pose a safety concern and low Columbic efficiency issue owing to the uncontrolled dendrite growth of Li and continuous SEI formation (side reactions). Furthermore, an excess of Li metal anode is used in most studies, which significantly decreases the practical energy density of Li−O_2_ batteries. We, therefore, present the research directions to achieve the reversible LiOH chemistry in Li−O_2_ batteries as follows.

1) Given the complexity of LiOH chemistry, we encourage systematic studies of reaction mechanisms by combining advanced experimental characterizations such as *in situ* surface-enhanced Raman spectroscopy and isotope-labeled DEMS with theoretical studies such as DFT calculations and MD simulations. In addition, it is necessary to study the effects of solvents, content of water, and catalysts on reaction mechanisms and electrochemical performance.
2) We encourage systematic studies on revealing the limiting factors that hinder the reversible oxidation of LiOH *via* 4e^−^ O_2_ evolution reaction, which will enable the rational design of effective redox mediators or solid catalysts. The reactive intermediates during discharge and charge should be identified, and their reactivity toward degradation of cell components should be carefully evaluated. Moreover, the isotope-labeled DEMS is highly recommended in order to evaluate the reversibility of LiOH chemistry to rule out the false O_2_ signal from Li_2_O_2_ decomposition or leaking cells.
3) We encourage the development of novel catalysts that can mitigate the reactive intermediates such as surface hydroxyl species while maintaining effective catalytic activity toward cleavage and reformation of O−O bond. A bio-inspired metal complex might be one of the promising catalysts for deactivating reactive intermediates toward reversible LiOH chemistry.
4) We encourage systematic studies of cycling Li−O_2_ batteries in ambient air. The effects of N_2_, CO_2_, and moisture on LiOH chemistry and Li metal anode should be carefully studied to fully address their detrimental effects.
5) We encourage the development of Li metal anode protection technologies to address safety issues and low Columbic efficiency caused by the uncontrolled dendrite growth of Li and continuous SEI formation. To be practical, a thin Li metal anode (small excess of Li) should be evaluated for advanced Li−O_2_ batteries.
